# Concurrent whole brain radiotherapy and bortezomib for brain metastasis

**DOI:** 10.1186/1748-717X-8-204

**Published:** 2013-08-21

**Authors:** Christopher D Lao, Judah Friedman, Christina I Tsien, Daniel P Normolle, Christopher Chapman, Yue Cao, Oliver Lee, Matt Schipper, Catherine Van Poznak, Daniel Hamstra, Theodore Lawrence, James Hayman, Bruce G Redman

**Affiliations:** 1 Department of Internal Medicine, University of Michigan, 1500 East Medical Center Drive, Ann Arbor 48109-0848, MI, USA; 2 University Hospitals, Cleveland, OH, USA; 3 Radiation Oncology, University of Michigan, Ann Arbor, MI, USA; 4 Department of Biostatistics, University of Pittsburgh, Pittsburgh, PA, USA; 5 Radiology, University of Michigan, Ann Arbor, MI, USA

**Keywords:** Radiation, Brain, Melanoma, Bortezomib, Phase I, TITE-CRM, Diffusion tensor imaging, MRI

## Abstract

**Background:**

Survival of patients with brain metastasis particularly from historically more radio-resistant malignancies remains dismal. A phase I study of concurrent bortezomib and whole brain radiotherapy was conducted to determine the tolerance and safety of this approach in patients with previously untreated brain metastasis.

**Methods:**

A phase I dose escalation study evaluated the safety of bortezomib (0.9, 1.1, 1.3, 1.5, and 1.7 mg/m^2^) given on days 1, 4, 8 and 11 of whole brain radiotherapy. Patients with confirmed brain metastasis were recruited for participation. The primary endpoint was the dose-limiting toxicity, defined as any ≥ grade 3 non-hematologic toxicity or grade ≥ 4 hematologic toxicity from the start of treatment to one month post irradiation. Time-to-Event Continual Reassessment Method (TITE-CRM) was used to determine dose escalation. A companion study of brain diffusion tensor imaging MRI was conducted on a subset of patients to assess changes in the brain that might predict delayed cognitive effects.

**Results:**

Twenty-four patients were recruited and completed the planned therapy. Patients with melanoma accounted for 83% of all participants. The bortezomib dose was escalated as planned to the highest dose of 1.7 mg/m^2^/dose. No grade 4/5 toxicities related to treatment were observed. Two patients had grade 3 dose-limiting toxicities (hyponatremia and encephalopathy). A partial or minor response was observed in 38% of patients. Bortezomib showed greater demyelination in hippocampus-associated white matter structures on MRI one month after radiotherapy compared to patients not treated with bortezomib (increase in radial diffusivity +16.8% versus 4.8%; p = 0.0023).

**Conclusions:**

Concurrent bortezomib and whole brain irradiation for brain metastasis is well tolerated at one month follow-up, but MRI changes that have been shown to predict delayed cognitive function can be detected within one month of treatment.

## Background

The development of metastatic disease to the brain often reflects a poor prognosis, with a median survival measured in months. Survival of patients with brain metastasis from historically more radio-resistant malignancies (i.e., melanoma and renal cell carcinoma) is particularly dismal. Of patients who develop symptomatic melanoma brain metastases, the metastases are fatal in up to 95% of cases [1]. Despite the recent improvements in treatment for metastatic melanoma death from melanoma brain metastasis continues to be a critical barrier to improved survival [2-4].

Whole brain radiotherapy (WBRT) can have an impact on CNS progression, neurologic decline, and the likelihood of death from cerebral metastases, but such treatment has not demonstrated an overall survival benefit as primary therapy [5]. Radio-sensitization has been attempted in melanoma but the results have been underwhelming [6]. The promise of such an approach, however, is still important to explore. Bortezomib (VELCADE®, Millennium Pharmaceuticals, Inc.) is a proteasome inhibitor with preclinical and clinical data supporting activity against a variety of neoplasms. For melanoma, there does not appear to be much activity as a single agent but its potential role as a radiosensitizer is promising [7-9]. Concurrent use of bortezomib and radiation to treat metastatic disease to the brain has not been assessed previously, thus, a phase I study of concurrent bortezomib and whole brain radiotherapy in untreated patients predominantly with melanoma was conducted.

## Methods

### Eligibility

Men and women aged 18 years of age or older with a histopathologically confirmed solid tumor malignancy and clinical evidence of metastatic disease to the brain were considered for enrollment. The study was initially designed only for patients with melanoma and renal cell carcinoma, but was later expanded to other solid tumors with documented brain metastases. The study was approved by the University of Michigan Institutional Review Board (IRB) and signed informed consent was obtained for study enrollment. Study participation required one or more brain metastases on contrast-enhanced brain MRI for which WBRT was a treatment option, and in the judgment of the treating physician beginning additional systemic therapy (e.g., chemotherapy) could wait at least 30 days from completion of WBRT. Patients who had brain metastases managed with radiosurgery or surgery in the past were eligible as long as they had not received WBRT. Other enrollment criteria included: an estimated survival of at least 8 weeks, Karnofsky performance score (KPS) of at least 70%, adequate hematologic, hepatic, cardiovascular and renal function, and completion of previous chemotherapy at least 2 weeks before starting WBRT. Females of child bearing potential must have had confirmation they were not pregnant and acceptable contraception was required for the duration of the study.

Patients were considered ineligible if they had received previous radiotherapy to the head or neck, WBRT, or prior bortezomib therapy. Female patients who were pregnant or breast-feeding or patients with ≥ Grade 2 peripheral neuropathy within 14 days before enrollment, class III or IV congestive heart failure or serious concurrent cardiac disease, hypersensitivity to boron or mannitol, or serious medical or psychiatric illness likely to interfere with participation were ineligible.

### Study procedures

Within seven days of registration, a complete history and general physical exam, including neurological examination was performed, as well as determination of KPS immediately prior to the beginning of protocol treatment. Documentation of steroid and anticonvulsant doses, complete blood count with differential and platelet count (CBCDP), and comprehensive chemistry profile were obtained. An MRI with gadolinium contrast was performed within 3 weeks prior to the start of therapy. During the treatment period, all participants were evaluated weekly by physical exam, including neurological examination and examination of the skin within the radiation treatment portal. Documentation of toxicities, KPS, and both steroid and anticonvulsant use were performed at each evaluation. A complete blood count with differential was performed prior to each dose of bortezomib. At the completion of WBRT, patients were evaluated once every two weeks for an additional month which completed study participation. Brain imagingwas obtained one month after completion of radiotherapy and then as clinically indicated approximately every 2 to 3 months. Following the first month post-irradiation, patients were seen and treated as clinically indicated.

### Radiation treatment

Adequate immobilization and reproducibility of position was ensured using thermoplastic masks. The target volume included the whole brain and meninges to the foramen magnum. Doses were specified at central axis at mid-plane. Radiation was delivered with a daily fraction size of 2.5-3.0 Gy per fraction given 5 days a week, for a total dose of 30–37.5 Gy. The Radiation Oncology treating physicians were allowed to determine the most appropriate treatment fractionation scheme (30 Gy in 3 Gy fractions or 37.5 Gy in 2.5 Gy fractions) for patients independent of bortezomib. Treatment was delivered using photon beam energies of 6 megavolts (MV).

### Bortezomib

Bortezomib for this study was provided by Millennium Pharmaceuticals, Inc. (Cambridge, Massachusetts). It is a small molecule proteasome inhibitor currently approved by the United States Food and Drug Administration and registered in Europe for the treatment of multiple myeloma. Bortezomib was given by rapid IV injection beginning on day 1 of radiation, at least one hour prior to radiation treatment. During the radiation therapy period, bortezomib was given twice per week (at least 72 hours apart) for a total of 4 doses (given on days 1, 4, 8, and 11).

### Dose assignment

The bortezomib dose was assigned using TITE-CRM method [10]. The primary objective of the study was to determine the dose of bortezomib associated with a 20% probability of DLT. A DLT was defined as the development of any ≥ grade 3 non-hematologic toxicity or ≥ grade 4 hematologic toxicity by National Cancer Institute Common Terminology Criteria for Adverse Events (CTCAE) version 3.0 that was possibly, probably or definitely attributed to bortezomib and/or WBRT identified up to thirty days following treatment. Before each drug dose, the patient was evaluated for possible toxicities that may have occurred after the previous dose(s). The trial planned to accrue 30 patients evaluable for toxicity.

As in all CRM and TITE-CRM trials, before the enrollment of the first patient, the expected probability of DLT at each dose was elicited from the clinical investigators by the statistician, who used this information to construct the prior estimate of the dose-toxicity function, a one-parameter logistic function. As each patient completed the trial, the dose-toxicity function was re-estimated, and the re-estimated function was used as patients presented for enrollment to calculate the estimated probability of DLT at each dose. Each newly enrolled patient was thereby assigned to the bortezomib dose with estimated probability of toxicity closest to but not exceeding the target rate, 0.20.

### Data analysis

The primary objective was to estimate the dose-toxicity function of bortezomib by means of a Bayesian logistic regression model, logit(π) = a + b × d, where π is the probability of toxicity, a and b are logistic regression parameters estimated from the dose and toxicity data, and d is dose of bortezomib. The secondary objective was to assess the response to the combination of bortezomib and WBRT by MRI or contrasted CT. Response criteria followed guidelines proposed by MacDonald et al. [11].

### Brain imaging

A companion University of Michigan IRB approved imaging study was conducted to assess MRI findings that might reflect changes in blood brain/tumor permeability in patients with brain metastases during and after radiotherapy. Patients enrolled in the Phase I bortezomib study were eligible to participate in the concurrent imaging study. Diffusion tensor imaging (DTI), which measures water diffusivity around axons and reflects the degree of demyelination, was used to evaluate a subset of patients enrolled to the concurrent WBRT-bortezomib study. All participating patients provided separate written informed consent for this study. Patients underwent brain MRI scans at three time points: 1–2 weeks pre-RT, within one week of finishing RT (end-RT), and one month after finishing RT (post-RT). All MRI scans were done on a single 3 T Phillips Achieva scanner. Functional Imaging Analysis Tool (FIAT), an imaging software package developed at the University of Michigan, was employed prior to Tract Based Spatial Statistics (TBSS). Details of these techniques and analysis were presented or published previously [12-15].

## Results

### Patient and treatment characteristics

Twenty-seven patients were screened for enrollment from January 29, 2007 to June 9, 2009. Twenty-four patients initiated and completed therapy on protocol, and represent the evaluable per-protocol sample. Enrollment was stopped at twenty-four despite the planned thirty as an adequate number of subjects had been accrued to the highest dose level. Three patients were considered ineligible due to either prior head and neck irradiation, rapid progression of disease or concomitant therapy that was not allowed. Twenty-three of the twenty-four patients (96%) received the planned four doses of bortezomib. Fifty-eight percent of patients were female and the median age was 58 years. The majority of patients had not received prior systemic therapy (67%). Patients with melanoma accounted for 83% of all participants. The median number of brain metastases was five and 50% of patients had neurologic symptoms related to disease at baseline (Table 1).

**Table 1:** Patient and treatment characteristics at baseline

**No. of patients**	
Screened	27
On-study	24
Gender, n (%)	
Female	14 (58)
Male	10 (42)
Median age, years (range)	58 (41–76)
Underlying malignancy, n (%)	
Melanoma	20 (83)
Non-small cell lung cancer	2 (8)
Renal cell carcinoma	1 (4)
Breast	1 (4)
No. previous systemic therapies	
Median (range)	0 (0–9)
No. of brain metastases	
Median (range)	5 (1- ≥ 10)
Neurologic symptoms at baseline, n (%)	
Yes	12 (50)
No	12 (50)
Dexamethasone use, n (%)	
Yes	17 (71)
No	7 (29)
Craniotomy^*^, n (%)	
Yes	2 (8)
No	22 (92)
Anti-epileptic medication use, n (%)	
Yes	2 (8)
No	22 (92)
Bortezomib dose (mg/m^2^/dose), n (%)	
0.9	5 (21)
1.1	4 (17)
1.3	4 (17)
1.5	2 (8)
1.7	9 (37)
Radiation dose, n (%)	
30 Gy (3 Gy fractions)	19 (79)
37.5 Gy (2.5 Gy fractions)	5 (21)

^*^One patient underwent craniotomy and was rendered disease-free in the brain after progressing on protocol therapy.

The bortezomib dose was escalated per-protocol via TITE-CRM from 0.9 mg/m^2^ to 1.7 mg/m^2^, with the largest cohort of patients (n = 9) receiving the highest dose level. A total radiation dose of 30 Gy was administered to 79% of patients and 37.5 Gy was administered to 21% of patients. All patients treated with 37.5 Gy (n = 5) were in the highest dose cohort (Table 1).

### Toxicities

**Figure 1:**
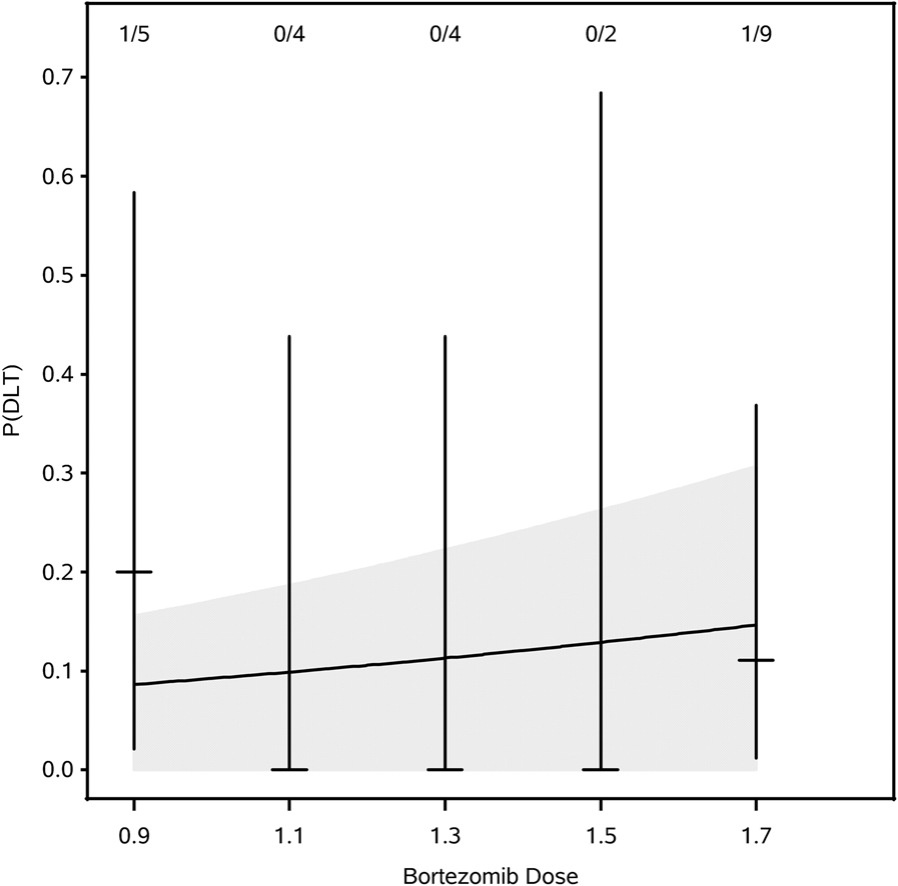
**Results of Markov Chain Monte Carlo estimation of dose-toxicity function.** Posterior estimate of dose-toxicity function (black curve) with one-sided, 90% credible region (gray area). Vertical lines are 90% one-sided exact confidence intervals for per-dose probability of toxicity (tick marks). Numbers at the top are #DLTs/# evaluable patients.

The relationship between bortezomib dose and DLT is depicted in Figure 1. The estimated P(DLT) at 1.7 mg/m^2^ is 0.15, with a one-sided 90% credible interval extending to 0.24. No grade 4/5 toxicities were observed that were related to treatment. The most frequent toxicity noted was fatigue (54% of patients) (Table 2). Two grade 3 DLTs were observed (hyponatremia and encephalopathy). Hyponatremia occurred in a patient treated at 0.9 mg/m^2^ and resolved after completion of protocol treatment. Encephalopathy developed in a patient treated at 1.7 mg/m^2^. This patient was admitted to the hospital for management prior to receiving all planned bortezomib doses. Symptoms were initially considered secondary to tapering of dexamethasone with improved symptoms following escalation of the steroid dose. Seizure activity could not be ruled out, but no antiepileptic medications were started. Immediately prior to the fourth bortezomib dose the patient developed expressive aphasia that prompted a second admission and precluded administration of additional bortezomib. Radiotherapy was completed as planned. Within four weeks of completion of protocol therapy the patient’s course was complicated by gastric perforation requiring surgery. This was considered possibly related to prolonged steroid exposure and stress ulcers. During that admission an electroencephalogram was performed and revealed diffuse slowing consistent with encephalopathy. Her symptoms did not significantly improve and she ultimately died from disease progression. This patient was not included in the companion brain imaging study.

**Table 2:** Adverse events possibly to definitely related to treatment

**Toxicity**	Bortezomib dose level*									
	**0.9 (n = 5)**		**1.1 (n = 4)**		**1.3 (n = 4)**		**1.5 (n = 2)**		**1.7 (n = 9)**	
	**Gr.1-2**	**Gr.3-4**	**Gr.1-2**	**Gr.3-4**	**Gr.1-2**	**Gr.3-4**	**Gr.1-2**	**Gr.3-4**	**Gr.1-2**	**Gr.3-4**
Neurologic										
Headache	2								4	
Neuropathy	1									
Confusion	1									
Memory impairment			1						1	
Tinnitus			1						1	
Speech impairment									1	
Muscle weakness									1	
Hearing impairment									2	
Seizure									1	
Encephalopathy										1^#^
Gastrointestinal										
Nausea/vomiting							1		5	
Diarrhea							1			
Laboratory										
Hyponatremia		1^#^								
Other										
Fatigue	3		2		3		1		4	
Alopecia	3				1		1			
Sweating	1									
Edema	1									
Mucositis			1						1	
Pain (scalp)							1		1	
Pain (extremity)									1	
Chills/rigors									1	
Skin										
Radiation dermatitis	1		3		4				1	
Dry skin	1									
Rash							1			
Pruritus					1					

^*^mg/m^2^/dose.

^#^Dose limiting toxicity.

### Response and survival

Twenty-one of twenty-four patients were evaluable for radiologic response in the brain. The three patients without adequate surveillance brain imaging had clinical evidence of progressive disease and continued on with supportive care alone that precluded repeat imaging. No complete responses were observed. A partial response (PR) in the brain was observed in 17% of patients, a minor response (mPR) in 21% of patients, and stable disease (SD) in 33% of patients. Partial responses and SD were observed across all cohorts. Survival data was available on all 24 patients. Median survival for the study population was 5 months (range 1.5 to 17 months) (Table 3). The relationships between dose and response and survival were not statistically significant, but the study was not formally powered for these endpoints.

**Table 3:** Response and survival

	**Best response in brain**^**#**^					**Median survival**
	**CR**	**PR**	**mPR**	**SD**	**PD**	**months (range)**
Study population (n = 24)^*^	0	4 (17%)	5 (21%)	5 (21%)	8 (33%)	5 (1.5–17)
Bortezomib dose group						
(mg/m^2^/dose)						
0.9 (n = 5)	0	1 (20%)	0	1 (20%)	3 (60%)	7 (5–11)
1.1 (n = 4)	0	2 (50%)	1 (25%)	1 (25%)	0	5.8 (2.5–7)
1.3 (n = 4)	0	0	3 (75%)	0	1 (25%)	9.5 (5–12.5)
1.5 (n = 2)	0	0	0	0	2 (100%)	11 (5–17)
1.7 (n = 9)^¶^	0	1 (11%)	1 (11%)	3 (33%)	2 (22%)	4 (1–8)
Radiation dose						
30 Gy (3 Gy fractions)	0	4 (100%)	4 (100%)	2 (25%)	7 (87.5%)	7 (1.5–17)
37.5 Gy (2.5 Gy fractions)^†^	0	0 (0%)	0	3 (75%)	1 (12.5%)	4 (1.5–8)

^*^ Pre- and post- treatment brain MRI or CT with contrast available on 22 patients.

^#^ CR: complete response; PR: partial response; mPR: minor response; SD: stable disease; PD: progressive disease.

^¶^ Pre- and post-treatment brain MRI or CT with contrast available on 7 of 9 patients. Survival data available on all patients.

^†^ Pre- and post-treatment brain MRI or CT with contrast available on 4 of 5 patients treated with 37.5 Gy.

### Brain imaging

**Figure 2:**
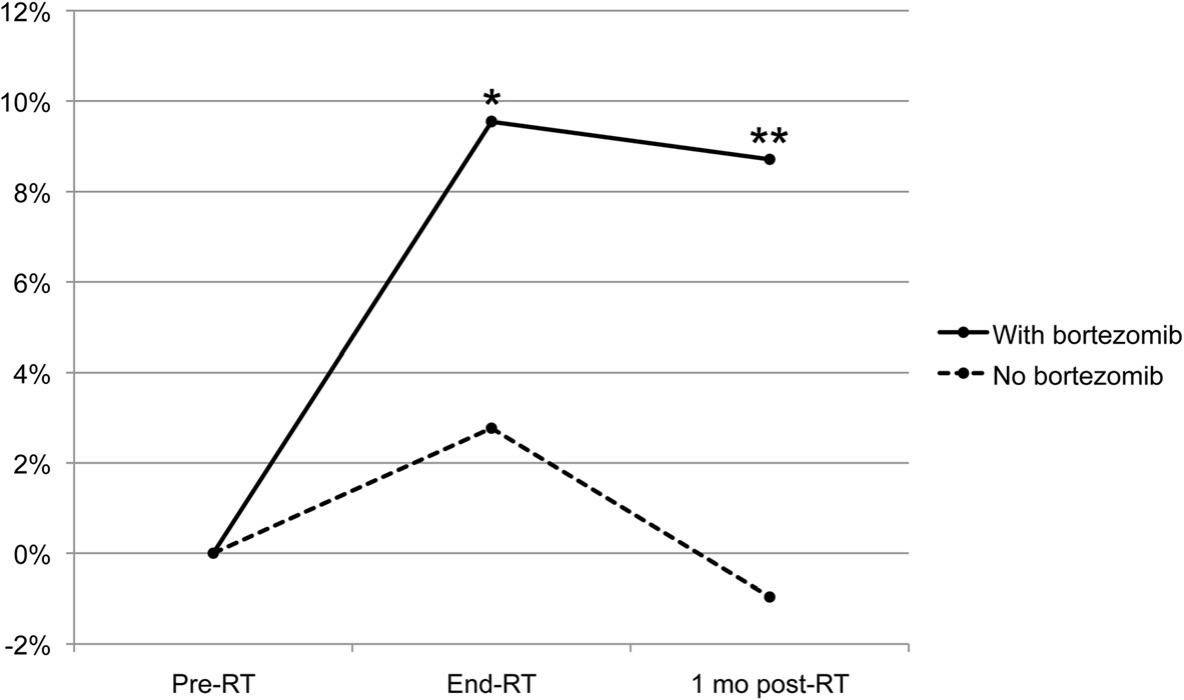
**Radial diffusivity changes with and without bortezomib in hippocampus-associated white matter.** Y-axis: Percent difference in radial diffusivity changes between hippocampus-associated and other white matter. *p < 0.05, **p = 0.0007 (for difference from 0%).

In the companion diffusion tensor MRI study to assess normal white matter radiation response, 23 patients with brain metastasis from any cancer treated with WBRT were enrolled. Twelve of those patients had completed at all three study MRIs (pre-RT, end-RT, post-RT) and met predetermined criteria for image quality, including no edema, mass effect, or metastases >5 mm diameter in the examined white matter tracts [13]. Of the 12 patients that completed imaging at one month post-RT, 8 (all with melanoma) received bortezomib on the phase I WBRT-bortezomib study and were compared to the other 4 patients who did not receive bortezomib. The four patients that did not receive bortezomib had cancer types other than melanoma. Of the 8 bortzemib patients, 2 received 37.5 Gy and 6 received 30 Gy. Of the 4 patients who did not receive bortezomib, 2 received 37.5 Gy and 2 received 30 Gy. In the 8 patients who received bortezomib, there was a significantly greater increase of radial diffusivity in hippocampus-associated white matter as compared to other white matter areas (Table 4). In the remaining 4 patients that did not receive bortezomib, there was no significant difference in the change of radial diffusivity between hippocampus-associated and other white matter structures. At the one month post-RT follow-up, there was a persistent greater increase in radial diffusivity in hippocampus-associated white matter in patients that received bortezomib than in patients that did not receive bortezomib (*p* = 0.0023; Figure 2).

**Table 4:** Diffusion tensor imaging bortezomib interaction effect. Changes in radial diffusivity (RD) values by treatment group and white matter structures

**Group**	**Structures**	Pre-RT to End-RT^*^		**Pre-RT to 1 month post-RT**^**#**^	
		**Change in RD**	**95% CI**	**Change in RD**	**95% CI**
No bortezomib	Other white matter	+ 10.4%	6.3 – 14.5%	+ 5.8%	1.1 – 10.5%
	Hippocampus-associated	+ 13.2%	7.5 – 18.8%	+ 4.8%	−1.4 – 11.1%
With bortezomib	Other white matter	+ 7.7%	4.6 – 10.8%	+ 8.1%	4.8 – 11.5%
	Hippocampus-associated	+ 17.2%	13.0 – 21.5%	+ 16.8%^¶^	12.4 – 21.2%

^*^Pre-RT to End-RT: total n = 14, n = 9 with bortezomib.

^#^Pre-RT to 1 month post-RT: total n = 12, n = 8 with bortezomib.

^¶^Comparison of changes in hippocampus-associated structures to other white matter structures p-value = 0.0023.

## Discussion

Bortezomib is a proteasome inhibitor with independent activity against various cancers and has demonstrated activity as a radiosensitizer in preclinical models [8]. Much of the preclinical work on proteasome inhibition and radiation sensitization has focused on the role of nuclear factor kappa B (NF- κB) [9]. In many cancer cell models, radiation treatment results in the activation of NF- κB which might act to inhibit apoptosis. In melanoma, however, as well as other cancer cell types, NF- κB is constitutively activated which could further mediate resistance to radiation therapy. Proteasome inhibition can result in the prevention of NF- κB activation, which may result in improved responses and outcomes. Other effects on DNA repair and survival pathways may also contribute, including suppression of homologous recombination, down-regulation of Bcl2, Bxl1, survivin, XIAP and increases in Fas/Fas ligand [16,17].

Data on concurrent bortezomib and radiotherapy is limited. Pugh et al. [18] performed a phase I study of bortezomib and radiotherapy for patients who were candidates for palliative radiation to sites other than central nervous system and bone. Doses up to 1.6 mg/m^2^ IV for 4 doses starting on day 1 of radiotherapy were given. No DLTs were observed. O’Neil et al. [19] performed a phase I study of 5-fluorouracil and bortezomib concurrent with RT for rectal cancer. Diarrhea was the dose limiting toxicity at the 1 mg/m^2^/dose level but NF- κB activation did not appear to be suppressed at that dose. Kubicek et al. [20] performed a phase I of concurrent bortezomib (up to 1.3 mg/m^2^/dose) and temozolomide with WBRT for primary central nervous system malignancies (85% had glioblastoma). Forty-four percent had previously received RT. Grade 3 headache, syncope, neuropathy, hyponatremia, dyspnea, stupor were observed but none were considered DLTs.

In our study of concurrent bortezomib and whole brain irradiation, we administered one cycle of bortezomib and observed only two DLTs up to 1.7 mg/m^2^/dose up to one month post treatment in previously untreated patients with metastatic cancer. The patient who developed grade 3 encephalopathy was symptomatic at baseline from multiple brain metastases, was the oldest patient enrolled (76 years old), and was heavily pretreated for her disease (breast cancer, estrogen receptor/progesterone receptor positive, HER2/neu negative). These factors may have contributed to the severity of this adverse event and it is unclear how much the bortezomib added to radiation contributed to the observed encephalopathy.

Phase I clinical trials involving brain irradiation for metastatic disease can be challenging and alternative strategies to adequately monitor patients are critical [21]. In this study, a Time-to-Event Continual Reassessment Method (TITE-CRM) design was used to optimize accrual with dose assignment determined by acute effects. Late radiation effects from brain irradiation, however, are exceptionally difficult to use as a dose limiting toxicity (DLT) in a phase I study. In the current study, the late radiation effects from irradiation were not formally assessed given the technical and practical difficulties in following a patient for months to determine toxicities prior to enrolling another patient. However, late effects are a critical part of assessing toxicities from brain irradiation as it is already recognized as a major issue in the pediatric survivorship population [22]. A number of investigators have attempted to assess delayed toxicities from radiation using surrogate imaging markers [23]. The understanding of the biologic causes of late cognitive decline are limited but evidence supports white matter loss resulting from demyelination and axonal degradation as key components which can be exploited as predictors of late cognitive impairment. Diffusion tensor imaging which assesses the diffusion of water around axons as a measure demyelination may be one of the best methods to determine this [24]. In the current study, the number of patients participating in the companion study with adequate follow-up was limited but the findings are strongly suggestive of white matter damage that did not recover four weeks after completion of RT for those who received bortezomib. No formal assessment of cognition was included in this study, but late cognitive impairment could be expected if patients survived longer [25]. With the expected improvement in therapeutic approaches for melanoma in particular, this issue will be critical [4].

Although bortezomib concurrent with WBRT did appear to have activity (59% had a PR, mPR or SD), which is historically better than what would be expected with WBRT alone for these typically radio-resistant malignancies, the median overall survival (5 months) was similar to what might be expected in this population at the time this study was conducted [6]. It is not possible from this study to quantify the contribution of bortezomib to WBRT.

## Conclusions

Bortezomib appears to be well tolerated up to 30 days after completion of administration of up to 1.7 mg/m^2^ when given concurrently with WBRT; however, changes in the normal brain were already evident at the end of RT and disease control appeared limited. Future studies testing concurrent brain irradiation with more promising therapeutic agents should employ techniques to monitor for changes in the normal brain that could predict delayed cognitive impairment.

## Competing interests

The author’s declare that they have no competing interests.

## Authors’ contributions

CDL, JF, CT, DPN, BGR were responsible for the study design and implementation. DPN, OL, and MS performed the data analysis. CT and YC designed and conducted the MRI imaging protocol. CVP, DH, TL and JH contributed to the implementation and manuscript writing. All authors read and approved the manuscript.
